# Systematic Review—The Potential Implications of Different Platelet-Rich Plasma (PRP) Concentrations in Regenerative Medicine for Tissue Repair

**DOI:** 10.3390/ijms21165702

**Published:** 2020-08-09

**Authors:** Pietro Gentile, Simone Garcovich

**Affiliations:** 1 Surgical Science Department, Plastic and Reconstructive Surgery, “Tor Vergata” University, 00133 Rome, Italy; 2 Scientific Director of AIRMESS, Academy of International Regenerative Medicine & Surgery Societies, 1201 Geneva, Switzerland; 3 Institute of Dermatology, F. Policlinico Gemelli IRCSS, Università Cattolica del Sacro Cuore, 00168 Rome, Italy; simgarko@yahoo.it

**Keywords:** PRP concentration, PRP tissue regeneration, PRP regenerative medicine, PRP in vivo effect

## Abstract

The number of studies evaluating platelet-rich plasma (PRP) concentration has substantially grown in the last fifteen years. A systematic review on this field has been realized by evaluating in the identified studies the in vitro PRP concentration—also analyzing the platelet amount—and the in vivo PRP effects in tissue regeneration compared to any control. The protocol has been developed in agreement with the Preferred Reporting for Items for Systematic Reviews and Meta-Analyses-Protocols (PRISMA-P) guidelines. Multistep research of the PubMed, MEDLINE, Embase, PreMEDLINE, Ebase, CINAHL, PsycINFO, Clinicaltrials.gov, Scopus database and Cochrane databases has permitted to identify articles on different concentrations of PRP in vitro and related in vivo impact for tissue repair. Of the 965 articles initially identified, 30 articles focusing on PRP concentration have been selected and, consequently, only 15 articles have been analyzed. In total, 40% (*n* = 6) of the studies were related to the fixed PRP Concentration Group used a fixed PRP concentration and altered the platelet concentration by adding the different volumes of the PRP (lysate) to the culture. This technique led to a substantial decrease in nutrition available at higher concentrations. Sixty percent (*n* = 9) of the studies were related to the fixed PRP Volume Group that used a fixed PRP-to-media ratio (*Vol/Vol*) throughout the experiment and altered the concentration within the PRP volume. For both groups, when the volume of medium (nutrition) decreases, a lower rate of cell proliferation is observed. A PRP concentration of 1.0 × 10^6^ plt/μL, appears to be optimal thanks to the constant and plentiful capillary nutrition supply and rapid diffusion of growth factors that happen in vivo and it also respects the blood decree-law. The PRP/media ratio should provide a sufficient nutrition supply to prevent cellular starvation, that is, PRP ≤ 10% (*Vol/Vol*) and thus best mimic the conditions in vivo.

## 1. Introduction

The number of studies evaluating platelet-rich plasma (PRP) concentration has substantially increased during the last fifteen years (2005–2020). PRP is a concentrate of platelets and growth factors (GFs) obtained by the centrifugation of venous blood. It may be autologous or allogenic and generally, it is possible to distinguish the autologous PRP in two different products—Autologous activated PRP (AA-PRP) and autologous not-activated PRP (A-PRP). These products are considered routine for plastic surgeons and dermatologists with consolidated experience in regenerative medicine [1,2]. Preparation methods are not standardized yet and regenerative mechanisms related to the biomolecular pathway are still the object of study. The in vivo/in vitro effects of AA-PRP and A-PRP in different field of regenerative medicine to improve the tissue repair have been reported [1,2,3]. In particular, in the last fifteen-years, the PRP application has expanded to a wide range of clinical fields, including plastic surgery [4], trichology [5,6,7], dermatology [8,9], maxillofacial surgery [10], orthopedics [11] and others.

The biomolecular rationale for the PRP’s application is represented by the regenerative properties of the platelets favoring the wound healing. Platelets hold about 50–80 alpha-granules that contain hundreds of bioactive proteins, including a wide range of GFs principally represented by platelet-derived growth factor (PDGF), fibroblast growth factor (FGF), vascular endothelial growth factor (VEGF), epidermal growth factor (EGF), transforming growth factor-beta 1 (TGF-β1), insulin-like growth factor (IGF), connecting tissue growth factor (CTGF) and hepatocyte growth factor (HGF) [12,13]. Every one of these major GFs is involved in a specific biomolecular function during tissue repair playing a fundamental role in wound healing and also in hair re-growth. In fact, when secreted, the GFs binding to the transmembrane receptors of the targeted area cells, producing cell growth, mitogenesis, chemotaxis, promoting the proliferation and extracellular matrix (ECM) formation and thus tissue repair [12,13].

Additionally, the A-PRP’s and AA-PRP’s anti-apoptotic effects have been described as the most important factors stimulating hair re-growth through the Bcl-2 protein activation (anti-apoptotic regulator) and Akt signaling, improving the dermal papilla cells survival (DPCs) during the hair cycle [2].

Currently, several kit and procedures are described, to prepare PRP. 

These procedures are different on the basis of the centrifugation’s time and g-force or revolutions used (revolutions per minute -RPM-), the platelet’s amount obtained, the GF type and amount and chemokine release. These differences are increased by wide biological and temporal variation [11]. Consequently, it seems to be very difficult to select which methods, kits and procedures for PRP are better [14] or which are more or less adequate for treating different types of tissue damages displaying different platelet counts and different PRP concentrations.

However, the positive impact and efficacy of PRP in tissue repair appear to be confirmed by the many studies published but a standardized procedure that aims to obtain the same platelet count in different PRP products has not yet been established. Additionally, different PRP concentrations added to the cell culture may produce different effects in terms of cellular proliferation and differentiation.

In the present systematic literature review, the effectiveness of different platelet concentrations for cell proliferation focusing on in vitro studies was evaluated, discussing the most effective PRP concentration in vivo.

## 2. Methods

### 2.1. Regulatory Framework on Clinical PRP Use

The human autologous PRP is considered a blood component for not-transfusional use. These blood components must be distinguished in two different categories—a) for infiltrative/topical use, (not considered cells therapy) b) for cellular therapy, which involves complex techniques of bioprocessing of therapeutic cells. In this last case, reference is made to Regulation n.1394/2007 of the European Parliament (EC) and by the reflection paper on the characterization of cutting edge treatment medicinal products draft concurred, 20 June 2014 European Medicines Agency (EMA)/Committee for Advanced therapies (CAT)/600280/2010 Rev 1, while for infiltrative/topical use the European references are represented by Decrees of November 9, 2007, n. 207 and n. 208 and each nation provide a national law.

Currently, in Italy, the PRP procedure must be performed following the dispositions reported in the “Blood Law-Decree, 2 November 2015” represented by several parameters that must be respected and obtained during PRP preparation as following displayed:

- 1 × 10^6^ µL ± 20% final platelet amount;
- Topical and/or infiltrative use exclusively;
- Quality and sterility checks on the sample obtained;
- Not over 55 mL of blood to withdraw for every patient;
- Labeling of every PRP sample;
- Specific Informed consent;
- Side events module;
- Data processing module;
- Patients screening on the basis of exclusion criteria (platelet disorders, thrombocytopenia, anti-aggregating therapy, bone marrow aplasia, uncompensated diabetes, sepsis and cancer).

PRP preparation methods must be conducted in accordance with the European Laws and Institutional Guidelines and they must be performed following the principles outlined in the Declaration of Helsinki and internationally consented ethics in clinical research [15], performing a quality assessment based on the Strengthening the Reporting of Observational Studies in Epidemiology (STROBE) checklist [16] and respecting the national laws representing in Italy by “Blood Law-Decree, 2 November 2015.”

For all physicians that want to start with the PRP applications in the regenerative medicine field, the authors suggest strictly respecting the guidelines highlighted in the Italian Blood Law-decree, as it was reported to follow all the Good Clinical Practices (GCPs). 

This article has been realized on the basis of a research contract (R. D. #1467/2017) between the first author P.G. and the University of Rome “Tor Vergata,” Italy.

### 2.2. Search Strategy

This systematic review was registered in International prospective register of systematic reviews (PROSPERO, https://www.crd.york.ac.uk/prospero/#myprospero) with ID code number: CRD42020196804.

Due to the growing interest in regenerative medicine via autologous biotechnologies, a number of studies have been conducted to assess the in vitro concentration of A-PRP and AA-PRP and related in vivo impact in tissue repair. A systematic review protocol was developed in accordance with the Preferred Reporting for Items for Systematic Reviews and Meta-Analyses-Protocols (PRISMA-P) guidelines. The search was conducted in accordance with the PRISMA guidelines and the Cochrane handbook. A multistep search of the PubMed, MEDLINE, Embase, PreMEDLINE, Ebase, CINAHL, PsycINFO, Clinicaltrials.gov, Scopus and Cochrane databases was performed to identify studies on different concentrations of PRP in vitro and related effects in vivo for tissue regeneration. Tissue regeneration with PRP searching without a language or publishing-time restriction has been performed. 965 articles (539 articles found in PubMed and 426 in EMBASE) using the keyword “PRP concentrations,” “Platelet concentrations in PRP products,” “PRP concentration and tissue regeneration,” “PRP implication in stem cell proliferation,” “PRP implication in stem cell differentiation” were identified.

Of the 965 articles initially identified, 30 articles focusing on PRP concentration were selected and, consequently, only 15 articles were analyzed.

#### 2.2.1. Study Assessment

The objective of the present systematic review was to assess the selected articles comparing the in vitro different PRP concentrations’ effects in stem cell proliferation and differentiation with the in vivo related impact on tissue regeneration. Additional objectives were to evaluate the impact of the different platelets and GFs concentration, in soft and bone tissue repair.

Investigations included in this article had to match predetermined criteria according to the PICOS (patients, intervention, comparator, outcomes and study design) approach. Criteria for inclusion and exclusion are specified as following—P-Patients (inclusion criteria—age 18–80 years, males and females who suffered from soft tissue defects, bone tissue defects, scars, outcomes of scars, burns, outcomes of burns, hemifacial atrophy, aging, ulcers, diabetic foot, lower extremity ulcers, chronic ulcers, skin disease, knee disease, cartilage disease, craniomaxillofacial disease, hair loss; and exclusion criteria—patient with platelets disorders, thrombocytopenia, anti-aggregating therapy, bone marrow aplasia, uncompensated diabetes, sepsis, cancer. No ethnicity restrictions were placed on the population of the study; I-Intervention (inclusion criteria—local and/or topical application of autologous PRP, autologous PRP infiltration, allogeneic PRP use, PRP in vitro analysis, PRP in vivo analysis.; exclusion criteria—sonicated PRP, pre-clinical investigations, animal studies related to not human transplants.); C-Comparator (inclusion criteria—any type of control, internal, external and different product; exclusion criteria—not provided); O-Outcomes (inclusion criteria—wound healing, soft tissue repair, bone tissue repair, cartilage repair; exclusion criteria—not applied); S-Study design (inclusion criteria—randomized clinical studies, randomized placebo-controlled trials/randomized, double-blind, placebo- and active-controlled, half-head trial/double-blind, placebo-controlled pilot trial/blinded, reviews, systematic review and metanalyses; exclusion criteria—case report, expert opinion, letter to editor, pre-clinical model (animal studies), experimental studies, investigations reporting PRP combined with biomaterials, articles identified as bias (not correct match with the key word used, group of study < 10 patients. No limitations were provided regarding the PRP method of preparation.

This systemic review, performed with the PICOS approach, in which only randomized placebo-controlled trials/randomized, double-blind, placebo- and active-controlled, half-head study/double-blind, placebo-controlled pilot study/blinded, randomized clinical trials, review, systematic review and metanalyses focused on PRP concentration in tissue regeneration were analyzed, is considered an EBM 1a level study according the Oxford Centre for Evidence-Based Medicine (OCEBM), March 2009 (https://www.cebm.net/2009/06/oxford-centre-evidence-based-medicine-levels-evidence-march-2009/).

#### 2.2.2. Study Selection

**Scheme 1:**
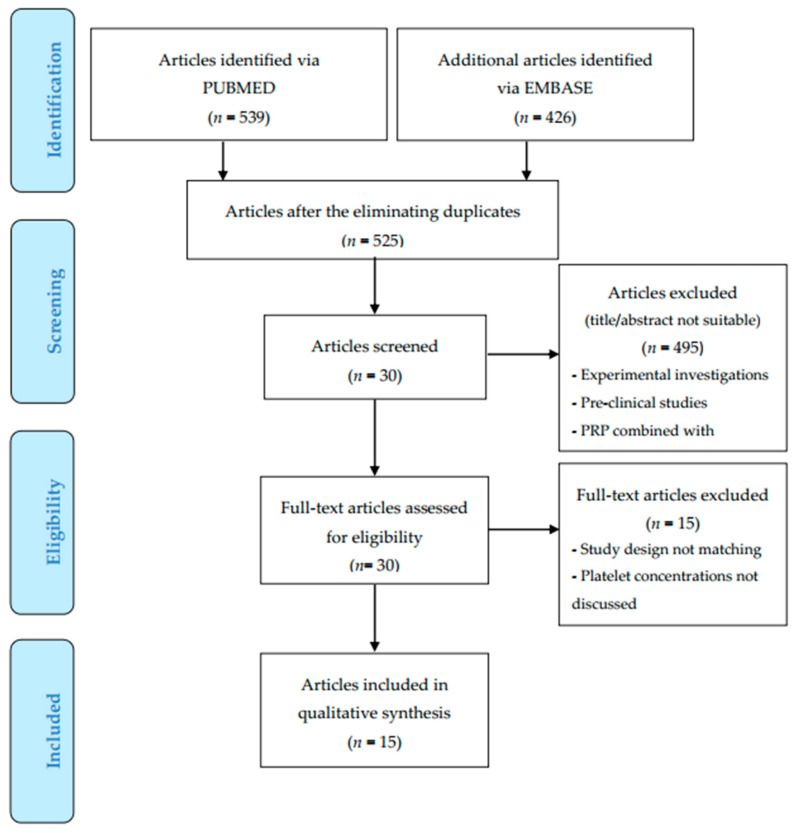
Preferred Reporting for Items for Systematic Reviews and Meta-Analyses (PRISMA) flow diagram.

In total, 965 records were identified and selected using Prisma Flow [17] (www.prisma-statement.org) (Scheme 1)—426 studies in EMBASE OvidSP, 539 in PubMed. The 160 duplicates in the two bases were excluded; the initial number of studies was 525. 

After the first screening (title and abstract), 495 studies were excluded, frequently due to the use of animal cells, no focus on different PRP concentration or because the design combined PRP with different types of biomaterials. Additional records were identified in the references. 

The remaining 30 papers were included for full-text screening, of which 15 papers were included for the final analysis.

#### 2.2.3. Data Extraction

Data were independently gathered by the first investigator (PG) and checked by the last investigator (SG), only from the retrieved articles. Any disagreement on the gathered data has been settled by a consensus between SG and PG. No attempt has been made to obtain specific or missing data from the investigators. The following data have been extracted—study design, patient number, first author, year of publication, type of procedure and primary and secondary results.

The quality of the included articles has been independently assessed by the first and the last investigators (PG and SG) using the Cochrane Collaboration’s Risk of Bias Assessment tool for RCT15 while using the Newcastle–Ottawa Scale to evaluate the individual non-randomized studies [18].

#### 2.2.4. Result Measures

The primary result has been the in vitro effect of different PRP concentrations in stem cell proliferation and differentiation (culture, microscopical and histological evaluation were analyzed). Secondary results were in vivo evaluation of PRP application/infiltration/topical use in tissue repair. The in vivo analysis was performed considering results of laboratory test, magnetic resonance imaging (MRI) and/or Computed Tomography (CT) of soft/bone tissue treated and biopsy. Objective evaluation (performed by the investigators and supported by instrumental analysis), subjective evaluation (performed by the patients treated on the basis of the healing, aesthetic and functional outcomes), a photographic and functional assessments were considered.

All results gathered from the articles have been reported with the same measurements retrieved from the papers. Advanced dressing, adipose-derived mesenchymal stem cells (AD-MSCs), Stromal Vascular fraction cells (SVFs) and autologous regenerative therapies have been used as a control in some of the included papers, while in other studies, patients were respectively allocated into study groups when they underwent PRP and to the control group when they underwent the placebo or other treatments. Missing data have been dealt with according to previously validated estimations [19,20].

## 3. Results

### 3.1. The In Vitro Impact of Platelet-Rich Plasma

In general, all the studies consistently report that PRP may increase cell proliferation in vitro. An exception is skin fibroblasts in the study of Anitua et al. [21]. Different studies assayed different extracellular substances and gene expression and thus difficult to compare systematically. Still, three studies observed an increased synthesis of collagen type I and/or II or hyaluronic acid (HA) [21,22,23]. Four studies observed increased cell motility and invasion at moderate [24,25,26] or relatively high concentration [27].

This systematic review detected two main types of groups:

- The Fixed PRP Concentration Group:

In a percentage of 40% (*n* = 6) of the studies analyzed, a fixed initial PRP concentration was used. Different concentrations were achieved by varying the PRP-to-media ratio (*Vol/Vol*) in which the cells were cultured. Mishra et al. [28] and Wang et al. [29] had a similar initial PRP concentration (1.0 and 1.55 × 10^6^ plt/μL, respectively). They found an increase in proliferation up to 10% PRP and a decrease when a volume of 20% PRP was used [28,29]. Hsu et al. [30] and Tavassoli-Hojjati et al. [31] used an initial PRP concentration of 1.124 and 1.194 × 10^6^, respectively and observed a maximal proliferation at 5% PRP. However, in these studies, a volume of 10% was not a part of the coarse test scale and when the next concentration was tested (15% and 50% PRP), the proliferation rate declined [30,31]. Amable et al. also observed an increase in cell proliferation up to 10% PRP and a decline when higher volume concentrations were used. The fixed PRP concentration was 2.94 ± 1.9 × 10^6^ plt/μL [32]. Giusti et al. [24] seem to belong to this group but do not clarify the PRP/media ratio (*Vol/Vol*) and thus it is difficult to compare to the others. It is difficult to compare the studies in an accurate metric manner due to the different initial PRP concentrations and different PRP volume concentrations tested. However, the data of this group loosely indicate that 10% of PRP (*Vol/Vol*) at a platelet concentration of 1–1.5 × 10^6^ is the most effective in vitro. In general, the technique of this group leads to a substantial decrease in nutrition available at higher concentrations—when the PRP concentration (volume) increases, the culture medium (nutrition) will decrease.

- The Fixed PRP Volume Group:

Sixty percent of the studies (*n* = 9) utilized a fixed ratio of PRP to culture media (*Vol/Vol*) throughout the experiment, for example, 10%/90% or 20%/80%. Different concentrations were achieved by diluting the original PRP-lysate within the chosen volume of PRP, frequently in Dulbecco’s Modified Eagle Medium (DMEM) or α-MEM. Haynesworth et al. [33], Jo et al. [22] and Wang et al. [29] used a ratio of 10% PRP (*Vol/Vol*) and observed an optimal proliferation rate at 1.6 × 10^6^, 4.0 × 10^6^ (fibroblast) and 1.5 × 10^6^ plt/μL, respectively. Similar results found by Rughetti et al. [27]. Anitua et al. [21] and Berger et al. [26], on the other hand, used a PRP volume of 20% and found maximum proliferation rate at 0.767 ± 0.95 and 0.875 (young group) × 10^6^ plt/μL, respectively— about half of Haynesworth et al. [33] and Wang et al. [29]. Graziani et al. [25] used the highest PRP/media ratio (33%/67%) and observed maximum proliferation of approx. 0.570 × 10^6^ (osteoblast) and 0.228 × 10^6^ (fibroblast) plt/μL. Unfortunately, Rughetti et al. [27] and Sadoghi et al. [34] did not clarify the exact ratio, which prevents a full comparison in the group. Sadoghi et al. [34] found the optimal platelet concentration to be five-fold and if we assume an average baseline in whole blood (WB) of 0.25 × 10^6^ plt/μL, the maximum proliferation can be estimated to be 1.25 × 10^6^ plt/μL [34]. When Haynesworth et al. [33] increased the PRP-to-media ratio to 20%/80% (*Vol/Vol*) to obtain a platelet concentration of 10-fold (3.2 × 10^6^ plt/μL), they observed a decrease in the proliferation rate.

One trait that emerges in this group is that studies using a fixed volume of PRP higher than 10% (*Vol/Vol*) observed lower optimal platelet concentration for proliferation. Still, Mazzocca et al. [35] differs from this trait and found that relatively moderate platelet concentrations as most effective, even if a PRP/media ratio of 10%/90% was used.

All the data analyzed has been showed in Table 1.

#### 3.1.1. Platelet-Rich Plasma Impact Modulated by Cell Type

This systematic review indicates that the effect of PRP varies according to cell type. According to Mishra et al. [28], Human Mesenchymal Stem Cells (HMSC) were more responsive to PRP than fibroblasts. Within the frame of the same experimental design, Graziani et al. [25] observed that the osteoblast was more receptive to a higher PRP concentration than fibroblasts. According to Jo et al. [36], the maximal proliferation rate for fibroblasts and HMSC was 4 × 10^6^ and 8 × 10^6^, respectively. The cell site origin might also be a factor. Anitua et al. [21] showed that fibroblasts from the skin did respond equally to PRP and Platelet poor Plasma (PPP), while PRP significantly stimulated the proliferation of fibroblasts from synovium and tendon in a dose-dependent manner. Regarding tendonal fibroblasts, earlier studies also report that tendons from different sites have different tissue structures, composition, cell phenotypes and metabolic characteristics [37,38]. Further research is needed to elucidate how different PRP concentrations affect different types of cells and how the same type of cells harvested from different sites respond.

#### 3.1.2. PRP-to-Culture-Media Ratio: A Potential Bias

The authors hypothesize that the main course of this difference is due to variations in the PRP-to- culture-media ratio (*Vol/Vol*). As seen in The Fixed PRP Concentration Group, the highest PRP concentrations implied significantly lower nutrition levels in the culture wells because of the diluting technique. This point is seen in Hsu et al. [30], Amable et al. [32] and is particularly obvious in the study of Tavassoli-Hojjati et al. [31]. In the latter case, PRP (not lysate) corresponding to a platelet concentration of 1,194,000/μL was mixed with DMEM to process PRP concentrations of 0.1%, 5% and 50% in which the cells were cultured. The optimal concentration of 5% contained 45% more nutrition than the PRP of 50%. A comparable pattern is seen in Giusti et al. [24], who initially produced a PRP that contained 4.5 × 10^6^ to 6 × 10^6^ plt/μL. To make different concentrations of PRP-lysate, the initial PRP-lysate was diluted in DMEM + 1% Fetal Bovine Serum (FBS). Then, starved cells were exposed to (i.e., cultured in) the different PRP concentrations. Although a statistical assessment is not possible, the optimal PRP-lysate of 0.5 × 10^6^ plt/μL had significantly more nutrition than the highest concentration (5 × 10^6^ plt/μL) [24].

The comparable finding is revealed in The Fixed PRP Volume Group. The studies that advocate moderate platelet concentrations as most effective in inducing cell proliferation tended to have a higher fixed volume of PRP and, thus, a lower volume of culture media throughout the experiment. Graziani et al. [25] used 100 μL media and 50 μL PRP of any concentrations, which means a PRP part of 33.3% (*Vol/Vol*). The PRP-lysate containing 2.5× and 3.5× was diluted in extra DMEM to obtain the appropriate concentration. The highest concentration—PRP-max (4.2–5.5×)—received no extra DMEM. The cell cultures treated with lower platelet concentrations (2.5× and 3.5×) received substantially more nutrition than the group of PRP-max. This point is also seen in Anitua et al. [21] and Berger et al. [26], who kept the PRP-to-media ratio constant at 20% over 80% and observed a platelet concentration of 0.767 ± 0.95 and 0.875 (young group) × 10^6^ plt/μL as most effective—approximately half of what is described in Haynesworth et al. [33], Rughetti et al. [27] and Sadoghi et al. [34]. Compared to Haynesworth et al. [33], Jo et al. [36] and Wang et al. [29] who utilized a 10/90 ratio (*Vol/Vol*), Graziani et al. [25] and Anitua et al. [21] used about 23% and 10% less nutrition, respectively.

#### 3.1.3. Platelet-Rich Plasma Concentration Analysis

PRP volume over 10% might be critical in vitro. We have already seen that optimal platelet concentration in both Graziani et al. [25] and Anitua et al. [21] was significantly lower than in those who utilized a PRP concentration of 10% (*Vol/Vol*). Mishra et al. [28] diluted PRP-lysates of 1 million plt/μL to MDEM at concentrations of 0.1%, 1% 5%, 10% and 20% (*Vol/Vol*) and observed the highest cell proliferation in the 10% PRP media. When 20% of PRP was used, they observed a significant decline in proliferation in the fibroblast culture. Comparable results were seen in Amable et al. and Haynesworth et al. when 20% PRP was used [32,33] and Hsu et al. when the PRP concentration was increased from 5% to 15% [30]. However, Chen et al. deviates from this prominent tendency and observed maximal proliferation when 5%, not 10%, PRP was applied to culturing dental pulp stem cells [39]. These findings are of importance, taking into consideration the dramatic increase in nutrition requirement that happens when a cell undergoes growth and proliferation compared to its quiescent state [40]. A large quantity of nucleotides, amino acids and lipids are required when a cell goes from the S0 state into the anabolic phases and proliferates into two viable daughter cells [41]. Therefore, there is reason to speculate that the PRP-to-media ratio in the studies that advocated a low or moderate platelet concentration resulted in a lack of cellular nutrition and possible starvation.

#### 3.1.4. Cell Density Per Well

The cell density per well (volume) may also be a bias factor, since an initial higher number of cells will require more nutrition for a prolonged proliferation and viability. Amable et al. [32] and Tavassoli-Hojjati et al. [31] utilized a 24-wells plate in their tests but seeded 1 × 10^3^ and 50 × 10^3^ cells/well, respectively. This may partly explain that the optimal platelet concentration according to Amable et al. [32] is 5–6 times higher than that according to Tavasolli-Hojjati et al. [31] (10% of 2.9 ± 1.9 × 10^6^ plt/μL versus 5% of 1.194 × 10^6^ plt/μL, respectively). With the exception of Amable et al. [32] and Graziani et al. [25], the included studies give no exact information about the cell density per well. In general, information about the type of culture plate (most often “96-wells plate”) and the number of cells/well or cells/cm^2^ is provided. However, the working volume inside each well is stretchable and allows for nutrition variation in each experiment. Therefore, the studies do not allow statistical analysis of this issue.

### 3.2. In Vivo Impact of Platelet-Rich Plasma

In vitro studies should always be utilized with care. However, since the studies reviewed above aimed to point out or suggest an ideal platelet concentration in PRP treatment, the optimal platelet concentration in vivo should be discussed. According to Vander Heiden et al. [41], the cells in multicellular organisms, including mammals, are normally exposed to a constant flow of nutrition supply. When PRP is injected in a tissue site and GFs are released, the arterial and capillary system will provide a constant flow of nutrition. The in vivo situation is, therefore, markedly different from the in vitro condition in which the nutrition supply is fixed and limited. In vivo, the GFs will also gradually be diffused and transported out of the target area relatively quickly, which leads to a decline in levels of GFs within hours, even if the molecular weight of the GFs is high (6–150 kDa) [42]. Based on these perspectives, the high concentration group seems to best mimic the conditions in vivo due to the low PRP-to-culture-media (nutrition) ratio. Still, extremely high platelet concentrations could be inadequate in injection sites where capillary density is low. Dernek et al. [43] tested the effect of PRP clinically on knee osteoarthritis at 1 million plt/μL (group 1) and 3 million plt/μL (group 2). Regarding Western Ontario and McMaster University’s Osteoarthritis Index (WOMAC) scores, both groups improved significantly but no significant difference was seen between the groups. Even if this study is based on small sample size and is not randomized, it indicates that PRP of 1 million plt/μL could be sufficient in intra-articular injections due to the low capillary density in these sites. Further investigation is required to elucidate these aspects regarding PRP-treatment. If we assume as a working hypothesis that the PRP/media ratio of 10% (*Vol/Vol*) is adequately imitating the condition in vivo and adjust the numbers accordingly, the optimal PRP concentration for cell proliferation the consequence of this ratio. The median of 1.55 × 10^6^ plt/μL indicates that a PRP concentration of 1.5 plt/μL might function as a working hypothesis for further research in vitro or in vivo. 

On the other hand, Cervelli et al. [4,44,45,46,47,48,49,50] reported the application of PRP in Plastic Surgery for the treatment of soft and bone tissue defects. In particular, they tested, for the first time in the literature, the in vitro impact of different PRP concentrations on AD-MSCs proliferation evaluating the related effects on in vivo tissue repair. Additionally, they evaluated the different percentages of fat graft maintenance enhanced with different PRP concentrations [4,45,50]. Regarding the in vitro test performed, they seeded 5000 AD-MSCs/cm^2^ in twenty-four-well plates incubating for twenty-four hours in DMEM containing 10% FBS [4,49]. At the end of this time, finished after twenty-four hours, the medium has been substituted with DMEM +10% FBS (control) and DMEM + 10% FBS + 5% PRP (study) [4,49]. After two, four, six and eight culture days, cells have been digested with 0.25% trypsin solution and then counted using a hemocytometer, with a trypan-blue exclusion [4]. The outcomes displayed a mean value of triplicate samples [4,49]. In detail, PRP produced a four-fold increase of AD-MSC number, at four and six days, when cells were at pre-confluency (*p* < 0.02) in morphological changes absence. After eight days, at confluency, there has been a three-fold increase of AD-MSCs number in PRP culture (study) versus control. Additionally, as mentioned above, they evaluated the related PRP dose-dependent impact in vivo, analyzing the wound healing in patients affected by the loss of substance [46,48], lower extremity ulcers [47] and the percentage of fat graft maintenance when used as lipofilling technique [45,50]. In detail, when the authors added 0.5 mL–0.4mL of PRP per each mL of fat, they observed that 61.1% and 88.9% of chronic lower extremity ulcers displaying a 100% reepithelization after 7.1 and 9.7-weeks, respectively, compared with 40% and 60% of the control group treated with HA. When 0.3 mL and 0.2 mL of PRP per each mL of the fat were added, 55.5% and 72.2% (for 0.3 mL) and only 44.4% and 66.6% (for 0.2 mL) respectively, of chronic lower extremity ulcers displayed the 100% re-epithelization during the same follow-up. They also observed in patients suffered by soft tissue defects treated with the combined use of fat grafting and PRP at a concentration of 0.5 mL or 0.4 mL per each ml of fat tissue, a 70% of fat graft maintenance one year later the treatment, observed only in 31% of the control group treated with the fat graft in absence of PRP addition; when they used 0.3 mL and 0.2 mL of PRP per each mL of fat tissue, they observed a 62% and 50% fat graft maintenance after one year. However, different cell types and tissue sites are factors that may up- or downregulate the optimal PRP concentration. Further research, in vitro and in vivo, is required to conclude this question.

### 3.3. Suggested Guidelines for Further Research

As shown, two main types of procedure have been utilized—The Fixed PRP Concentration Group leads to severe variation in nutrition availability in the culture. On the other side, The Fixed PRP Volume Group kept the nutrition level relatively constant throughout the experiment by limiting the variation within a smaller ratio. The latter group should be considered the most accurate for in vivo conditions. The exact number of cells per well and the volume of the well are factors that should be appreciated since an initial smaller number of cells/mL would require less nutrition to proliferate compared to a high number.

Based on the above discussion, the following basic guidelines for further in vitro studies are recommended.

- The PRP/media ratio should be kept fixed throughout the experiment to minimize nutritional variations at different PRP concentrations.
- The PRP/media ratio should provide a sufficient nutrition supply to prevent cellular starvation, that is, PRP ≤ 10% (Vol/Vol). This implies that the initial PRP concentration should be high since an increase of concentration by increased volume is not recommended.
- The cell density (cells/mL) should be defined, that is, both the number of cells per well and nutrition volume should be clarified.

### 3.4. Limitations

This systematic review has several limitations that may pose a risk of possible biases:

Research in the last two decades strongly suggests that not only platelet concentration but also other biological qualities are crucial in regard to the effect of PRP [51]. Especially the role and number of white blood cells (WBCs) [52] and levels of fibrinogen/fibrin [53] have been highlighted as important co-elements. This review has not incorporated these aspects since several studies have not metrically clarified other biological properties of the PRP than platelet concentration.

Since the blood and the cultured cells came from different donors, the experiments in most of these studies were not autologous. Even if only PRP-lysate was used, graft versus host reactions are a possible bias. This point should especially be appreciated in the studies of Tavassoli-Hojjati et al. [31], who used PRP (not lysate), Mazzocca et al. [35] because of the high number of WBCs and Wang et al. [29] who used a diffusion chamber (DC). These important aspects fall outside the scope and frame of this review. Also, different activation method [26,31,36], pH condition [28] and the choice of anticoagulant [54] have been thematized in the literature as co-elements that can increase or decrease the effect of PRP and thus may pose risks of biases to this review.

One of the principal aspects revealed by the present systematic review has been the absence of a standardized protocol both for PRP preparation (kit vs. methods and procedures) and PRP injection (manual vs. mechanical and controlled) as well as a standardized evaluation method absence. The absence of standardization of these parameters making it more difficult to assess the effectiveness of PRP among the different investigations. Regarding the several and different procedures of PRP preparation, a lack of consensus regarding the choice of preparation method has been revealed, it influenced by centrifugation timing, g-force and RPM used, platelet concentration, blood volume gathered and amount of PRP injected. Treatment protocols also varied in the number of sessions, the time interval among procedures, the anticoagulant use and the follow-up period analyzed.

## 4. Discussion

The in vitro studies here reviewed state almost consistently that PRP stimulates the proliferation of the human cell. This observation is also the case regarding cell motility and exocytosis of several important regenerative extracellular ground substances, for example, collagen type I and III, HA and so forth. Regarding the optimal platelet concentration for cell proliferation, the studies diverge severely. The review reveals two main types of experimental techniques utilized—Group 1 used a fixed PRP concentration and altered the platelet concentration by adding the different volumes of this PRP-lysate to the culture. Group 2 used a fixed PRP-to-media ratio (*Vol/Vol*) throughout the experiment and altered the PRP concentration in the fixed PRP volume. An overall trait is seen—When the PRP concentration increases, the volume of culture media (nutrition) decreases and a lower optimal concentration for cell proliferation is observed. This is particularly prominent in group 1, The Fixed PRP Concentration Group, due to the diluting technique. We hypothesize that the techniques used by some groups led to adverse growth conditions and even cell starvation when high platelet concentrations were tested. Due to the constant nutrition supply and rapid diffusion of growth factors in all animals, the authors consider the studies that advocate a relatively high platelet concentration to best mimic the situation in vivo. Thus, the studies that advocate a low and moderate PRP concentration, for example, Graziani et al. [25], Giusti et al. [24] and Anitua et al. [21] are not supported. However, hyper-concentrations might also be inappropriate, especially in tissue types that are poorly vascularized. Based on the findings, the author suggests a platelet concentration of approx. 1.5 × 10^6^/μL as a working hypothesis for further research in vitro and in vivo. Still, other concentrations might be beneficial depending on cell type and tissue site. 

One of the problems most frequently raised by the scientific international audience is the level of consolidated evidence-based medicine (EBM) offered by PRP in the in vivo treatment of soft/bone tissue defects [10,11,45,48,50], aesthetic defects [55], wound healing [46,47,56,57] and hair loss [5,6,7,58,59,60,61] compared versus FDA-approved and/or EMA/CAT and/or other institutions. The rationale of this problem is borne by the fact that many institutional guidelines of several nations are based on the EBM impact of a drug/procedure. For this reason, the authors feel the necessity to contribute consolidation of the EBM of PRP use via this systemic review of randomized/controlled/clinical trials (identified as EBM level 1a study), evaluating the in vitro impact of different PRP concentrations on cell proliferation, the related in vivo effect, reporting the most updated information compared with the last systemic reviews and adding an EBM 1a study to this topic.

## 5. Conclusions

This systematic review suggests a positive impact of PRP on tissue repair, as shown by in vivo, present a safe and effective procedure. Regarding the in vitro evaluation, the optimal PRP/media ratio was PRP ≤ 10% (*Vol/Vol*) while the optimal platelet concentration since to start for effective PRP implication was 1.0–1.5 × 10^6^/μL.

Probably, the next future will be based exclusively on autologous and/or allogeneic regenerative based therapies and for this reason, it appears to be necessary to invite all the international audience to read the present work and related articles with the aim of promoting the publication of only EBM level 1–2 studies.

## Figures and Tables

**Table 1:** Synthetic analysis of the outcomes: platelet-rich plasma (PRP) concentration (plt/μL) and PRP Volume (*Vol/Vol*).

Investigators	PRP Concentration Evaluated (plt/μL or Fold or WB)	Optimal PRP Concentration Cell Proliferation (*Vol/Vol* and plt/μL or Fold)	Optimal PRP Concentration Cell Motility and Invasion (plt/μL)	Cells Type Evaluated	Fixed PRP Concentration Proliferation/(plt/μL)	Optimal PRP/Media Ratio (*Vol/Vol*) for Cell Proliferation	Procedure
Wang et al. (2019) [29]	Platelet lysate corresponding to 0.2 × 10^6^, 0.5 × 10^6^, 0.8 × 10^6^, 1.0 × 10^6^, 1.2 × 10^6^, 1.5 × 10^6^, 2.0 × 10^6^, 2.7 × 10^6^ and 3.0 × 10^6^	10% of 1.5–3.0 × 10^6^	Not tested	HMSCs (bone marrow)	1.55 × 10^6^	10%	1.25, 1.5 and 1.9 × 10^6^ (mean 1.55 × 10^6^) has been added to the culture at a ratio of 1%, 5% and 10% to media (*Vol/Vol*)
Hsu et al. (2009) [30]	1.124 × 10^6^ plt/μL has been added to the wells in concentration of 2%, 5%, 15%, and 30%	5% of 1.124 × 10^6^ of 5% (*Vol/Vol*)	Not evaluated	Fibroblast, Osteoblast	1.124 × 10^6^	5%	PRP of 1.124 × 10^6^ plt/μL has been added to the wells in concentration of 2%, 5%, 15% and 30%
Mishra et al. (2009) [28]	1 × 10^6^ plt/μL (not-activated) has been added to the media at ratio of 1%, 5%, 10% and 20% (*Vol/Vol*)	10% of 1 × 10^6^	Not evaluated	Fibroblast, HMSCs	1 × 10^6^	10%	A standardized PRP containing 1 × 10^6^ plt/μL has been added to the culture at a ratio of 0.1%, 1%, 5%, 10% and 20% (*Vol/Vol*)
Chen et al. (2012) [39]	Lysate from PRP of 1. × 10^6^ has been added to the media at ratio of 0%, 1%, 5%, 10%, lysate (*Vol/Vol*)	5% of 1 × 10^6^	Not evaluated	Dental Pulp Stem Cells	1. × 10^6^ plt/μL	5%	PRP of 1. × 10^6^ plt/μL has been added to the media (α-MEM) at ratio of 0%, 1%, 5%, 10%, (*Vol/Vol*)
Tavassoli-Hojjati et al. (2016) [31]	1.194 × 10^6^ plt/μL diluted in DMEM resulting in concentration of 0.1%, 5% and 50%	5% of 1.194 × 10^6^	Not evaluated	Fibroblast	1.194 × 10^6^	5%	Initial PRP contained an average of 1.194.000 plt/μL
Giusti et al. (2014) [24]	0.5 × 10^6^, 1 × 10^6^, 2 × 10^6^, 3 × 10^6^ and 5 × 10^6^ plt/μL	0.5 × 10^6^ (PRP/media ratio unclear)	0.5 × 10^6^, at 46 h (PRP/media ratio unclear)	Tenocyte	4.5 × 10^6^ to 6 × 10^6^	Not displayed	Initial PRP contained 4.5 × 10^6^ to 6 × 10^6^ plt/μL. The cells have been treated with PRP-lysate which was diluted in culture medium + 1% FDS to obtain 0.5 × 10^6^, 1 × 10^6^, 2 × 10^6^, 3 × 10^6^ and 5 × 10^6^ plt/μL
Amable et al. (2014) [32]	2.94 +/− 1.9 × 10^6^ plt/μL has been mixed with culture media (α-MEM) to obtain the following PRP concentration: 1%, 2.5%, 5%, 10%, 20%, 30%, 40% and 50% (*Vol/Vol*)	10% of 2.94 +/− 1.9 × 10^6^	Not evaluated	HMSC (bone marrow, adipose tissue and Wharton’s Jelly)	2.94 +/− 1.9 × 10^6^	10%	PRP of 2.94 +/− 1.9 × 10^6^ plt/μL has been mixed with culture media (α-MEM) to obtain the following PRP concentration: 1%, 2.5%, 5%, 10%, 20%, 30%, 40% and 50% (*Vol/Vol)*
Haynesworth et al. (2002) [33]	0.625-fold, 1.25-fold, 2.5-fold and 5-fold	10% of 1.6 × 10^6^ (5-folds over baseline)	Not evaluated	HMSCs, Fibroblast	1.6 × 10^6^	10%	The initial PRP contained 1.6 × 10^3^ plt/μL (5-fold). PRP-lysates corresponding to PRP concentrations of 0.625, 1.25 and 2.5 has been made by diluting the lysate in DMEM. Each concentration has been added to the media in a ratio of 10%/90% (*Vol/Vol*)
Graziani et al. (2006) [25]	2.5-fold, 3.5-fold and 4.2–5.5-fold (PRP Max)	33.3% of 2.5 ≈ 0.570 × 10^6^	33.3% of 2.5 × Ca 0.570 × 10^6^	Osteoblast, Fibroblast	2.5 × 0.570 × 10^6^ (Osteoblast); 1 × 0.228 × 10^6^ (Fibroblast)	33%	Initial PRP contained 800.000–1.370.000 plt/μL. Maximum PRP-lysate (PRP-Max) has been diluted in DMEM to obtain PRP-lysate containing 250%, 350% over WB baseline
Rughetti et al. (2008) [27]	0.3 × 10^6^, 0.5 × 10^6^, 0.75 × 10^6^, 1.25 × 10^6^, 1.75 × 10^6^, 2.25 × 10^6^, 2.75 × 10^6^, 3.25 × 10^6^, 4 ×10^6^, 5 × 10^6^ and 7 × 10^6^	1.25 × 10^6^	1.5 × 10^6^	Endothelial cell	1.25 × 10^6^	Not displayed	Initial PRP has been activated and diluted in DMEM + 2.5% FCS (proliferation) or in DMEM only (motility and invasion). Platelet concentration at 3 × 10^5^, 5 × 10^5^, 7.5 × 10^5^, 1.25 × 10^6^, 1.75 × 10^6^, 2.25 x10^6^, 2.75 × 10^6^, 3.25 × 10^6^, 4 × 10^6^, 5 × 10^6^ and 7 × 10^6^ plt/μL has been added to the medium
Anitua et al. (2009) [21]	0.16 +/− 1 x10^6^, 0.404 +/− 39 x10^6^, 0.767 +/− 95 × 10^6^ plt/μL (2× and 4× of baseline)	20% of 0.767 +/− 95 × 10^6^ and 0.404 +/− 39 × 10^6^	Not evaluated	Fibroblast	0.767 +/− 95 × 10^6^	20%	200% and 400% of WB baseline. Platelet concentration of 404 +/− 39 × 10^3^ and 767 +/− 95 × 10^3^ added to media in a 20%/80% ratio
Mazzocca, (2012) [35]	PRP_LP_: 382.0 +/− 111.6 × 10^3^ plt/μL: PRP_DS_: 472.6 +/− 224.2 x10^3^ plt/μL; PRP_HP_: 940.1 +/− 425.8 × 10^3^ plt/μL	10% of PRP_LP_: 382.0 +/− 111.6 × 10^3^ plt/μL; 10% of PRP_DS_: 472.6 +/− 224.2 × 10^3^ plt/μL; No significance between PRP_LP_ PRP_DS_ and PRP_HP_	Not evaluated	Myocytes, Osteoblast, Tenocyte	382.0 +/− 111.6 × 10^3^ (PRP_LP_) − Myocytes 472.6 +/− 224.2 × 10^3^ (PRP_DS_)-Osteoblast; 382.0 +/− 111.6 × 10^3^ (PRP_LP_)-Tenocytes	10%	PRP_LP_: 382.0 +/− 111.6 × 10^3^ plt/μL: PRP_DS_: 472.6 +/− 224.2 × 10^3^ plt/μL; PRP_HP_: 940.1 +/− 425.8 × 10^3^ plt/μL
Wang et al. (2011) [23]	1.2–1.9 × 10^6^/μL, 1%, 5% and 10% (*Vol/Vol*)	10% of 1.5 × 1.9 × 10^6^ (4×)	Not evaluated	Tenocyte	1.55 × 10^6^	10%	1.25, 1.5 and 1.9 × 10^6^ (mean 1.55 × 10^6^) has been added to the culture at a ratio of, 1%, 5% and 10% to media (*Vol/Vol*)
Sadoghi et al. (2013) [34]	1-, 5- and 10 fold PRP has been obtained by diluting initial PRP in PPP. The PRP/media ratio is unclear	5-fold ≈ 1.25 × 10^6^	Not evaluated	Fibroblast	5-fold	Not evaluated	1-, 5- and 10 fold PRP has been obtained by diluting initial PRP in PPP. The PRP/media ratio is unclear
Berger et al. (2019) [26]	Platelet lysate corresponding to platelet levels of 14×, 7×, 3.5×, 1.75× and 0.9× of WB	20% of 0.875 × 10^6^ (Young group); 20% of 3.5 × 10^6^ (Old group)	Old group: 3.5 × 10^6^	Fibroblast	Young group: 0.875 × 10^6^ Old group: 3.5 × 10^6^	20%	Platelet lysate (PL) has been diluted in PPP to obtain lysates corresponding to platelet levels of 14×, 7×, 3.5×, 1.75× and 0.9× of WB

## Abbreviations

PRP	Platelet-Rich Plasma
PPP	Platelet-poor plasma
A-PRP	Autologous-Non-activated Platelet-Rich Plasma
AA-PRP	Autologous-Activated Platelet-Rich-Plasma
GFs	Growth Factors
VEGF	Vascular endothelial Growth factors
PDGF	Platelet Derived Growth factors
IGF-1	Insulin like Growth factor-1
TGF-ß	Transforming growth factor-beta
EGF	Epidermal growth factor
DPCs	Dermal papilla cells
EC	European Committee
CAT	Committee for Advanced treatments;
GMP	Good Manufacturing Practices
GCP	Good Clinical Practices
ECM	Extracellular matrix
SVFs	Stromal vascular Fraction Cells
AD-MSCs	Adipose-derived Mesenchymal Stem Cells
RPM	Right per minute
PRISMA-P	Preferred Reporting for Items for Systematic Reviews and Meta-Analyses-Protocols
CTGF	Connecting tissue growth factor
EBM	Evidence Based Medicine
MRI	Magnetic resonance imaging
CT	Computed Tomography
HA	Hyaluronic acid
OCEBM	Oxford Centre for Evidence-Based Medicine
WOMAC	Western Ontario and McMaster University’s Osteoarthritis Index scores
WBCs	White blood cells
DMEM	Dulbecco’s Modified Eagle Medium
FBS	Fetal Bovine serum
PLT	Platelets
